# Therapeutic Potential of Targeting Stromal Crosstalk-Mediated Immune Suppression in Pancreatic Cancer

**DOI:** 10.3389/fonc.2021.682217

**Published:** 2021-07-05

**Authors:** Wenting Du, Marina Pasca di Magliano, Yaqing Zhang

**Affiliations:** ^1^ Department of Surgery, University of Michigan, Ann Arbor, MI, United States; ^2^ Rogel Cancer Center, University of Michigan, Ann Arbor, MI, United States; ^3^ Department of Cell and Developmental Biology, University of Michigan, Ann Arbor, MI, United States

**Keywords:** pancreatic cancer, tumor microenvironment, immune suppression, T cells, myeloid cells, cancer-associated fibroblasts

## Abstract

The stroma-rich, immunosuppressive microenvironment is a hallmark of pancreatic ductal adenocarcinoma (PDA). Tumor cells and other cellular components of the tumor microenvironment, such as cancer associated fibroblasts, CD4^+^ T cells and myeloid cells, are linked by a web of interactions. Their crosstalk not only results in immune evasion of PDA, but also contributes to pancreatic cancer cell plasticity, invasiveness, metastasis, chemo-resistance, immunotherapy-resistance and radiotherapy-resistance. In this review, we characterize several prevalent populations of stromal cells in the PDA microenvironment and describe how the crosstalk among them drives and maintains immune suppression. We also summarize therapeutic approaches to target the stroma. With a better understanding of the complex cellular and molecular networks in PDA, strategies aimed at sensitizing PDA to chemotherapy or immunotherapy through re-programing the tumor microenvironment can be designed, and in turn lead to improved clinical treatment for pancreatic cancer patients.

## Introduction

Pancreatic ductal adenocarcinoma (PDA) is the most common form of pancreatic cancer, the third leading cause of cancer related death in the United States, with a 5-year survival rate of around 10% (1, 2). Over 80% of PDA patients are diagnosed at a late stage when the tumor is already locally advanced or metastatic to distant organs and therefore do not qualify for surgery (1). Standard chemotherapy, such as Gemcitabine alone or in combination of Albumin-bound paclitaxel (Abraxane) has long been the standard of care for PDA. However, it provides only modest survival benefit since a large percentage of patients are either intrinsically resistant or develop resistance soon after treatment starts (3). Multidrug regimens such as FOLFIRINOX (combination of oxaliplatin, irinotecan, leucovorin, and fluorouracil) have become standard of care for those patients that can tolerate it, as they increase patient median overall survival to 11.1 months. However, FOLFIRINOX is associated with high toxicity (4). Therefore, there is urgent need for the development of novel therapeutic strategies for PDA patients. Immune checkpoint blockade has achieved significant therapeutic success for a subset of cancer patients. Unfortunately, single agent immunotherapy has been ineffective in PDA (5). The reasons for this failure are complex, and likely stem from the nature of the stroma-rich tumor microenvironment (TME) in PDA, with abundant immunosuppressive cells such as cancer associated fibroblasts (CAFs) (6), CD4^+^ T cells (7) and myeloid cells including tumor associated macrophages (TAMs) and myeloid derived suppressor cells (MDSCs) (8–11). The stromal and immune compartments are linked by a web of interactions that promotes immune evasion of PDA cancer cells and contributes to the onset and progression of pancreatic carcinogenesis, affecting cell plasticity, metastasis, chemo-resistance and radiotherapy-resistance (3, 7, 12–18). This review summarizes the crosstalk between several key cell types that are dominant within the immunosuppressive TME of pancreatic cancer and discusses the most promising immune regulatory approaches to activate anti-tumor immune responses in PDA.

## The Immunologically “Cold” TME Is Modulated by Oncogenic Pathways in PDA

PDA initiates with activating mutation of oncogenes such as *KRAS* (mutant in over 90% of tumors, and present in the majority of precursor lesions as well) (19) and followed by inactivation of tumor suppressors such as *CDKN2A* or *P53* (altered in 90% and 70% of PDAs, respectively) (20–23). Pancreatic cancer develops from precursor lesions such as pancreatic intraepithelial neoplasia (PanIN) that over time progress to advanced and metastatic stage (24). Other types of precursor lesions such as intraductal papillary mucinous neoplasms and mucinous cystic neoplasms (25) are less common and have been reviewed elsewhere (20). Genetically engineered mouse models (GEMMs) that harbor pancreas-specific expression of oncogenic *Kras*, such as KC (*LSL-Kras^G12D^; p48/Pdx-1-Cre*) (26) and iKras* model (*p48-Cre; R26-rtTa-IRES-EGFP; TetO-Kras^G12D^*) (27), recapitulate the stepwise carcinogenesis process of human PDA. PanIN occurs spontaneously in these models and can progress to metastatic cancer with long latency. Using the KC model, researchers discovered that immunosuppressive cells, including CD4^+^ FOXP3^+^ regulatory T cells (Tregs), TAMs, and MDSCs, accumulate both in PanIN and PDA stages compared to normal pancreas (28). CD8^+^ cytotoxic T cells are scarce in PanIN and only present in a subset of PDA; even when they are present, they lack effector function (28). Similar kinetics of leukocytic infiltration were also described in the more aggressive KPC (*Kras^LSL-G12D/+^*; *Trp53^LSL-R172H/+^*; *Pdx-1-Cre*) GEMM (29). Reduced infiltration of CD8^+^ cytotoxic T cells and increased infiltration of CD4^+^FOXP3^+^CD25^+^ Tregs in PDA have also been shown in human patient samples (30, 31). The evidence from both GEMMs and patient samples indicate an immunologically ‘cold’ TME of PDA. Even when CD8^+^ T cells are present within the tumor nest in a small cohort of PDA patients, they are dysfunctional or exhausted (32). Recently, our laboratory defined an exhausted CD8^+^ T cell phenotype in human PDA by expression of T cell immunoglobulin and ITIM domains (TIGIT), an immune checkpoint that is relatively understudied (11). Using a combination of mass cytometry, single-cell RNA sequencing (scRNA-seq) and multiplex immunohistochemistry, we found increased markers of CD8^+^ T cell dysfunction with an up-regulation of TIGIT in PDA compared to non-malignant pancreas samples; further, the dysfunctional status of CD8^+^ T cells was more pronounced at later stages of carcinogenesis (11).

Oncogenic *KRAS* is a key mediator of immune suppression in PDA. A recent study using scRNA-seq approaches and TCGA data analysis suggest greater immune infiltration in KRAS independent and KRAS-low tumors compared to KRAS dependent and KRAS-high groups (33). In this model, inactivation of mutant *Kras* in PDA cells did not affect their tumorigenic capacity, but led to failure to evade the host immune system (33). The authors determined that KRAS knockout (KO) PDA cells had a striking up-regulation of major histocompatibility complex I (MHC I) genes compared with KRAS intact control cells, underlying increased susceptibility to anti-tumor immunity. M1-like TAMs, CD8^+^ cytotoxic T cells and natural killer T (NK T) cells dominated in KRAS KO tumors. Mechanistically, this study identified BRAF and MYC as key downstream regulators of KRAS-driven tumor immune suppression for PDA maintenance (33). MHC I accumulation in the cell is also negatively regulated by autophagy, which is in turn activated by oncogenic KRAS (34).

**Figure 1:**
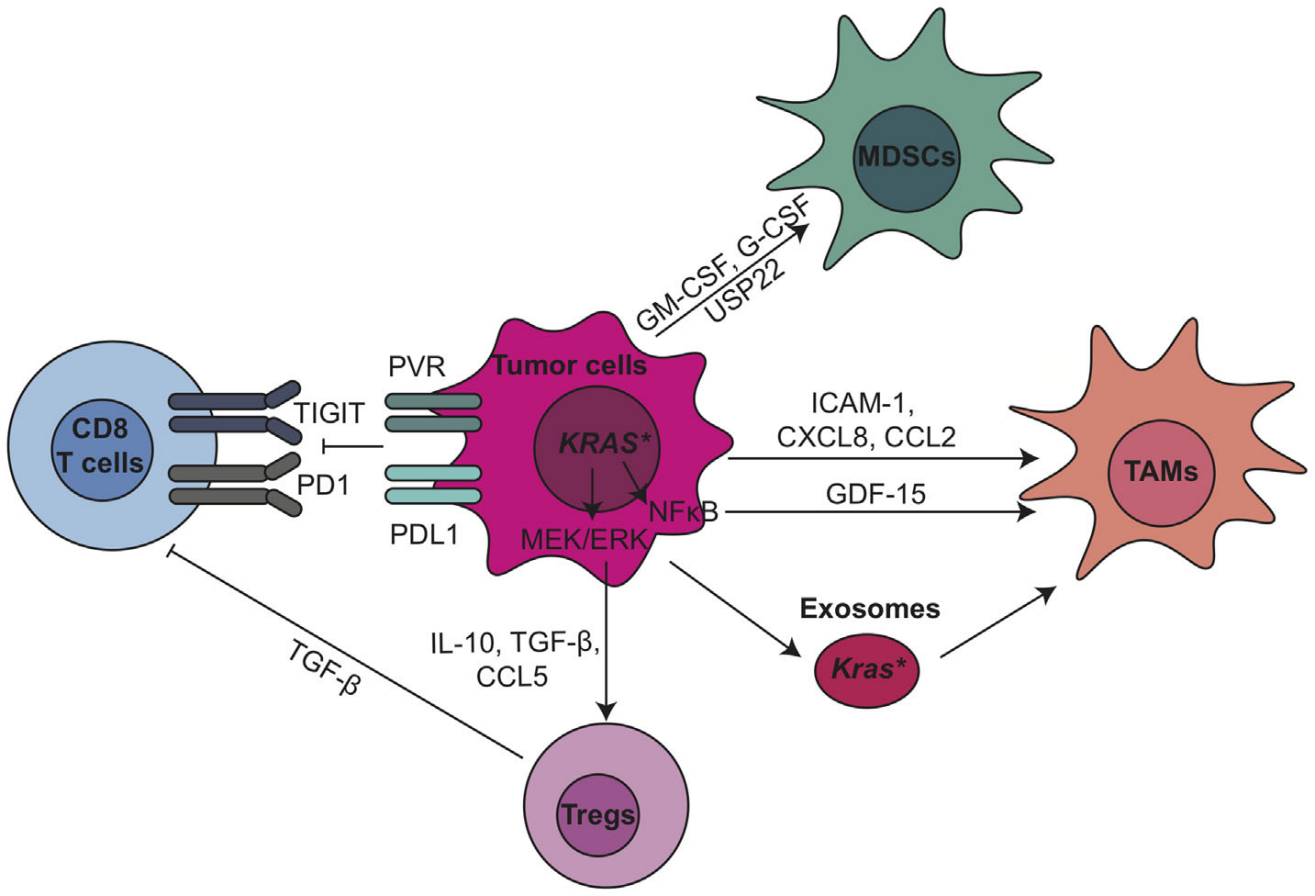
Tumor epithelial cells modulate immunosuppressive tumor microenvironment through oncogenic pathways and immune checkpoint pathways in PDA. CCL, C-C motif chemokine ligand; CXCL, C-X-C motif chemokine ligand; ERK, extracellular signal-regulated kinase; G-CSF, granulocyte-colony stimulating factor; GDF-15, growth/differentiation factor-15; GM-CSF, granulocyte-macrophage colony-stimulating factor; ICAM-1, intercellular adhesion molecule 1; MDSCs, myeloid-derived suppressor cells; MEK, mitogen−activated protein kinase kinase; NF-κB, nuclear factor kappa B; PD-1, anti-programmed cell death 1; PD-L1, programmed cell death ligand 1; PVR, poliovirus receptor; TAMs, tumor associated macrophages; TGF-β, transforming growth factor β; TIGIT, T cell immunoreceptor with Ig and ITIM domains; Tregs, regulatory T cells; USP22, ubiquitin specific peptidase 22.

KRAS activates essential pathways to control the expression and secretion of cytokines and chemokines from tumor cells, thereby regulating the recruitment and development of immune cells. For example, granulocyte–macrophage colony-stimulating factor (GM-CSF) produced by pancreatic cancer cells carrying the *KRAS^G12D^* mutation recruits immunosuppressive myeloid cells (35, 36). A key downstream effector of *Kras^G12D^* is the mitogen−activated protein kinase kinase (MEK)/extracellular signal-regulated kinase (ERK) pathway. Mitogen-activated protein kinases (MAPK)/ERK targets include interleukin-10 (IL-10) and transforming growth factor beta (TGF-β), which in turn induce Treg differentiation (37). MAPK/ERK signaling also induces expression of intercellular adhesion molecule (ICAM-1), which acts as chemoattractant for macrophages (38). Besides, growth and differentiation factor 15 (GDF-15), a direct target of nuclear factor kappa B (NF-κB) in tumor cells, suppresses the pro-apoptotic activity of macrophages by inhibiting tumor necrosis factor (TNF) and nitric oxide (NO) production (39). Depletion of GDF-15 in the KPC mouse model delayed tumor development and was accompanied by increased infiltrating antitumor macrophages (39). Extracellular *Kras^G12D^* in tumor-derived exosomes directly promotes alternatively activated or M2-like macrophage polarization *via* signal transducer and activator of transcription 3 (STAT3)-dependent fatty acid oxidation (40). Blocking *Kras^G12D^* release from tumor cells and uptake by macrophages suppresses macrophage-mediated pancreatic tumor growth *in vivo* (40). Other inflammatory mediators secreted by PDA cells include granulocyte colony-stimulating factor (G-CSF) (41), IL-6 (42), IL-1α (43), IL-1β (44, 45), ubiquitin specific peptidase 22 (USP22) (46), C-X-C motif chemokine ligand 8 (CXCL8) (47), matrix metallopeptidase 9 (MMP-9) and indoleamine-2,3-dioxygenase (IDO) (48), which all contribute to the establishment of immunosuppressive TME in pancreatic cancer (**Figure 1**).

PDA cells can also induce immune suppression by engaging critical immune checkpoint pathways such as programmed cell death protein 1 (PD1)/programmed death-ligand 1 (PD-L1). In addition, we recently discovered that Poliovirus receptor (PVR), one of the checkpoint TIGIT ligands, is expressed by tumor epithelial cells (11). Previously we showed that expression of PD-L1 in PDA cancer cells is regulated by epidermal growth factor receptor (EGFR)/MAPK signaling cascade (49). PD-L1 expressed by PDA cells directly induced the apoptosis of PD-1-expressing T cells, and its expression positively correlated with poor prognosis and inversely correlated with tumor-infiltrating T cells, particularly CD8^+^ T cells, in pancreatic cancer (50). These pre-clinical data have encouraged clinical trials targeting PD-1 and PD-L1, either as monotherapy or in combination with radiation or chemotherapy, in pancreatic cancer (**Table 1**, current active clinical trials, and **Table 2**, past clinical trials targeting pancreatic cancer TME). However, single agent immune checkpoint blockade has thus far been unsuccessful in PDA patients. One potential reason for this failure is the exclusion of active T cells from TME, which reveals an urgent need for strategies transforming the immunologically ‘cold’ microenvironment into ‘hot’. Targeting oncogenic signaling pathways such as KRAS, BRAF and MEK potentially provides an opportunity to alter the TME and sensitize PDA to immune checkpoint blockade (33, 51).

**Table 1:** Currently active clinical trials targeting the microenvironment of PDA.

Target	Agent	Combination	Identifier	Phase	Subjects
CCR2/5	BMS-813160	Chemotherapy or Nivolumab	NCT03184870	1/2	Pancreatic cancer
		GVAX, Nivolumab and SBRT	NCT03767582	1/2	Locally Advanced PDA
CD40	CDX-1140	Pembrolizumab, or chemotherapy	NCT03329950	1	Pancreatic adenocarcinoma
	Selicrelumab	Atezolizumab + Chemotherapy	NCT03193190	1/2	Metastatic pancreatic ductal adenocarcinoma
CSF1R	IMC-CS4	GVAX/CY and Pembrolizumab	NCT03153410	1	Pancreatic cancer
CTLA-4	Ipilimumab	Nab-Paclitaxel/Gemcitabine, Nivolumab and SBRT	NCT04247165	1/2	Locally advanced pancreatic cancer
	Ipilimumab + Nivolumab	Radiotherapy	NCT02866383	2	Pancreatic cancer/Metastastic pancreatic cancer
			NCT03104439	2	
			NCT04361162	2	
	Tremelimumab + Durvalumab	Minimally invasive surgical microwave ablation	NCT04156087	2	Non-resectable pancreatic cancer
CTLA-4 + LAG3	XmAb22841	Monotherapy/Pembrolizumab	NCT03849469	1	Pancreatic cancer
CXCR1/2	SX-682	Nivolumab	NCT04477343	(phase) 1	Pancreatic cancer
DC	DC Vaccine		NCT03592888		Pancreatic adenocarcinoma
			NCT04157127	(phase) 1	
			NCT04627246		
GM-CSF	GVAX/CY		NCT01088789	2	Pancreatic cancer
		Nivolumab	NCT02451982	1/2	Pancreatic cancer
		IDO1 inhibitor (Epacadostat), Pembrolizumab, and CRS-207	NCT03006302	2	Metastatic pancreatic adenocarcinoma
		Nivolumab and SBRT	NCT03161379	2	Pancreatic cancer
		CRS-207, Nivolumab, and Ipilimumab	NCT03190265	2	Pancreatic cancer
	OH2 (oncolytic virus expressing GM-CSF)		NCT04637698	1/2	Locally advanced/metastatic pancreatic cancer
IL-1β	Canakinumab	Spartalizumab, Nab-paclitaxel, and Gemcitabine	NCT04581343	1	Metastatic pancreatic ductal adenocarcinoma
IL-12	Oncolytic adenovirus expression IL-12	Standard chemotherapy	NCT03281382	1	Metastastic pancreatic cancer
IL-6	Siltuximab	Spartalizumab	NCT04191421	1/2	Metastatic pancreatic adenocarcinoma
	Tocilizumab	Nab-Paclitaxel and Gemcitabine	NCT02767557	2	Unresectable panreatic carcinoma
		Ipilimumab, Nivolumab and SBRT	NCT04258150	2	Pancreatic cancer
PD-1	Cemiplimab	Plerixafor	NCT04177810	2	Metastastic pancreatic cancer
		Motixafortide (CXCR4 inhibitor), Nab-paclitaxel, and Gemcitabine	NCT04543071	2	Pancreatic cancer
	Nivolumab	Losartan, Folfirinox and SBRT	NCT03563248	2	Pancreatic cancer
		Tadalafil and vancomycin	NCT03785210	2	Metastatic liver cancer from pancreatic cancer
		FT500 (iPSC-derived NK cell product)	NCT03841110	1	Pancreatic cancer
		Chemotherapy	NCT03970252	1/2	Resectable pancreatic cancer
		Stereotactic radiotherapy	NCT04098432	1/2	Locally advanced non-resectable pancreatic cancer
		Irreversible electroporation	NCT04212026	2	Metastastic pancreatic cancer
		SX-682 (CXCR1/2 inhibitor)	NCT04477343	1	Pancreatic ductal adenocarcinoma
	Pembrolizumab	Neoadjuvant chemoradiation	NCT02305186	1/2	Resectable pancreatic cancer
		CPI-006 (CD73 antibody)	NCT03454451	1	Pancreatic cancer
		SBRT	NCT03716596	1	Pancreatic cancer
		Defactinib	NCT03727880	2	Resectable pancreatic ductal adenocarcinoma
		Lenvatinib (VEGFR inhibitor)	NCT03797326	2	Pancreatic cancer
		GB1275 (CD11b modulator)	NCT04060342	1/2	Pancreatic adenocarcinoma
		NT-I7 (Efineptakin Alfa)	NCT04332653	1/2	Pancreatic cancer
		EGFR/TGFβ Fusion Protein BCA101	NCT04429542	1	Pancreatic cancer
PD-L1	Durvalumab	Stereotactic ablative body radiotherapy (SABR)	NCT03245541	1/2	Pancreatic adenocarcinoma
		Oleclumab (CD73 antibody) and chemotherapy	NCT03611556	1/2	Metastatic pancreatic adenocarcinoma
TGFβR1	PF-06952229		NCT03685591	1	Pancreatic neoplasms

Clinical trial identifier from https://clinicaltrials.gov. CCR, C-C motif chemokine receptor; CSF1R, colony-stimulating factor 1 receptor; CTLA-4, cytotoxic T-lymphocyte-associated protein 4; CXCR, C-X-C motif chemokine receptor; DC, dendritic cell; GM-CSF, granulocyte-macrophage colony-stimulating factor; GVAX, GM-CSF gene transduced irradiated prostate cancer vaccine cells; IDO1, indoleamine 2,3-dioxygenase 1; LAG3, lymphocyte activating 3; PD-1, anti-programmed cell death 1; PDA, pancreatic ductal adenocarcinoma; PD-L1, programmed cell death ligand 1; SBRT, stereotactic body radiation; TGFβR, transforming growth factor β receptor.

**Table 2:** Past clinical trials targeting the microenvironment of PDA.

Target	Agent	Combination	Identifier	Status	Results
BTK	ACP-196	Pembrolizumab	NCT02362048	Completed	Well tolerated, limited clinical activity
	Ibrutinib	Durvalumab	NCT02403271	Completed	Well tolerated
		Gemcitabine and Nab-Paclitaxel	NCT02562898	Active, not recruiting	Ineffective
CD40	CP-870,893	chemotherapy	NCT00711191	Completed	Partially effective
	RO7009789	Gemcitabine and Nab-Paclitaxel	NCT02588443	Completed	Acceptable toxicity and clinical activity
	APX005M	Gemcitabine and Nab-Paclitaxel with or without Nivolumab	NCT03214250	Active, not recruiting	Manageable toxicity and early efficacy
CSF1R	Pexidartinib	Durvalumab	NCT02777710	Completed	Acceptable toxicity
	Cabiralizumab	Nivolumab	NCT02526017	Completed	Partially effective
		Nivolumab	NCT03336216	Active, not recruiting	Ineffective
DC	DC vaccine		NCT03114631	Completed	Safe with early clinical efficacy
RIPK1	GSK3145095		NCT03681951	Terminated	Serious adverse events
TGFβR1	Galunisertib	Durvalumab	NCT02734160	Completed	Partially effective

Clinical trial identifier from https://clinicaltrials.gov. BTK, Bruton tyrosine kinase; CSF1R, colony-stimulating factor 1 receptor; DC, dendritic cell; Receptor-interacting serine/threonine protein kinase 1 (RIPK1); TGFβR, transforming growth factor β receptor.

## CD4^+^ T Cells and Their Crosstalk With Stromal Cells Negatively Regulate the Tumor Immunity in PDA

CD4^+^ T cells infiltrate into the pancreas starting at early stages (PanINs) of carcinogenesis (28). Genetic depletion of CD4^+^ T cells increased tumor infiltrating CD8^+^ T cells and up-regulated their capacity to produce IFN-γ and granzyme B, therefore inhibiting tumorigenesis in a GEMM of PDA in a CD8^+^ T cell-dependent manner (7). This highlights that the formation of immunosuppressive microenvironment occurs even at the onset of pancreatic tumorigenesis, and shows that CD8^+^ T cells mediated anti-tumor immunity during PDA initiation is negatively regulated by CD4^+^ T cells.

CD4^+^ T cells include several subtypes, such as T helper 1 (Th1) cells, T helper 2 (Th2) cells, IL-17-producing T helper (Th17) cells, and Tregs (10). Th1 cells secrete pro-inflammatory cytokines such as Interferon gamma (IFN-γ), IL-2, TNF-α, IL-8, and IL-1β and can have anti-tumor effects (52). In contrast, Th2 cells secrete anti-inflammatory cytokines, such as IL-4, IL-5, and IL-10, and are tumor-promoting (53, 54). In human PDA, Th2 (GATA-3^+^) cells are predominant over Th1 (T-bet^+^) cells and the ratio of Th2/Th1 is an independent predictive marker of reduced patient survival (54). CD25^+^ Th17 cells express high levels of cytotoxic T-lymphocyte-associated protein 4 (CTLA-4) and mediate CD8^+^ T cell suppression in an immune checkpoint dependent manner (55). IL-17 secreted by Th17 cells accelerates PanIN initiation and progression by acting directly on epithelial cells that express the IL17 receptor (56). IL17 also recruits neutrophils, triggers neutrophil extracellular traps and excludes cytotoxic CD8^+^ T cells from tumors (57). Thus, pharmacological and genetical inhibition of IL17/IL17RA signaling in the KPC model increased immune checkpoint blockade sensitivity (57).

**Figure 2:**
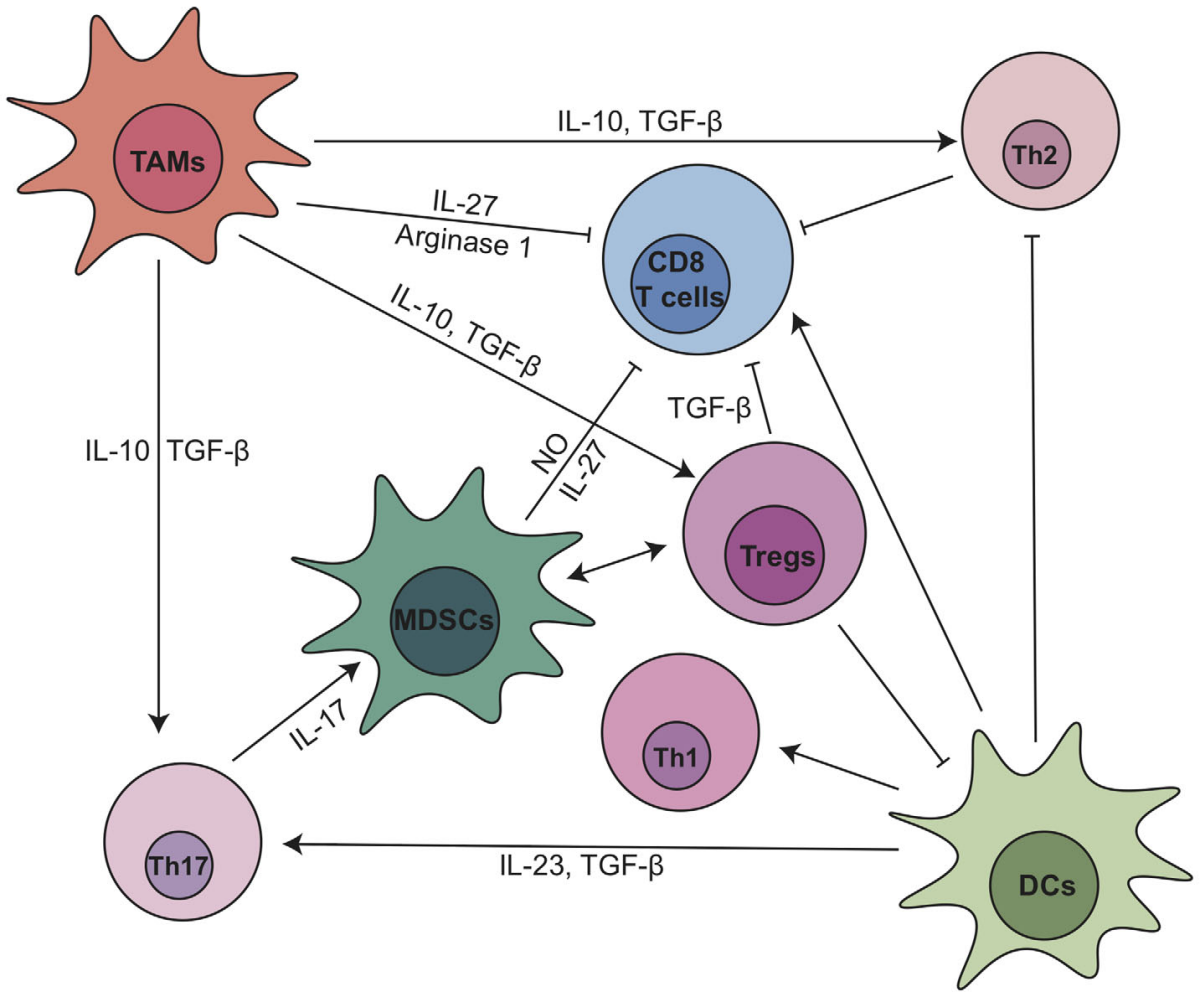
The dynamic cellular and molecular interactions between T cells and myeloid cell subsets in pancreatic cancer. DC, dendritic cell; IL, interleukin; MDSCs, myeloid-derived suppressor cells; NO, nitric oxide; TAMs, tumor associated macrophages; TGF-β, transforming growth factor β; Th, T helper; Tregs, regulatory T cells.

Tregs, defined as CD4^+^FOXP3^+^CD25^+^ T cells, are the most abundant CD4^+^ T cell subpopulation in PDA TME (28). High number of Tregs positively correlates with the progression and poor prognosis of PDA patients (31, 58). Tregs can be recruited by C-C chemokine ligand 5 (CCL5) (59). Disrupting CCL5/C-C chemokine receptor 5 (CCR5) signaling inhibited Treg migration to tumor (60). Tregs promoted the development of PDA through the suppression of IFN-γ-producing-CD8^+^ T cells in an orthotopic implantation model with primary Kras^G12D^-expressing pancreatic ductal epithelial cells (61). In this model, intratumoral Tregs directly interacted with tumor associated CD11c^+^ dendritic cells (DCs) and reduced their expression of costimulatory molecules necessary for CD8^+^ T cell activation such as CD40, CD80 and CD86 (61). Ablation of Tregs led to the restoration of immunogenic tumor-associated CD11c^+^ DCs and increased CD8^+^ T cell-dependent antitumor immunity, which resulted in an inhibition of tumor growth (61). Interactions between T cells and myeloid cell subsets are summarized in **Figure 2**, and we will further discuss their crosstalk within pancreatic cancer TME in Section 4.

Recently, a new study from our laboratory showed that Treg depletion failed to relieve immunosuppression and accelerated tumor progression in the KC and KPC GEMMs (62). Our study suggests that Tregs are a key source of TGFβ which facilitates the expansion of α-smooth muscle actin (αSMA)^+^ CAFs. Depletion of Tregs reprogramed the fibroblast populations inducing loss of tumor-restraining αSMA^+^ CAFs. The reprogramed fibroblasts secreted increased level of chemokines such as CCL3, CCL6, and CCL8 that act as chemoattractant for suppressive myeloid cells. Therefore, Treg depletion resulted in increased Arginase1 (Arg1)^+^ and PD-L1^+^ TAMs, restoring the immunosuppressive TME and promoting carcinogenesis. This effect was inhibited by an inhibitor for the common CCL3/6/8 receptor CCR1. Treg depletion also led to an increase in Th2 cytokine profile, indicating that the absence of Tregs fails to restore immune surveillance, likely because of compensation driven by other sub-populations of CD4^+^ T cells and immunosuppressive myeloid cells. Thus, a better approach might be reprogramming Tregs rather than depleting them altogether.

## Targeting Tumor Myeloid Cells as a Crucial Therapeutic Strategy To Relieve Immunosuppression in PDA

Myeloid cells, including immature myeloid cells (also commonly referred to as MDSCs), TAMs and tumor associated neutrophils (TANs) accumulate during the progression of pancreatic cancer (10). Myeloid cells directly promote acinar cell dedifferentiation during the earliest stages of pancreatic cancer (63). Our group has shown that myeloid cells are required for the establishment of an immunosuppressive environment in pancreatic cancer (49). EGFR ligands secreted by tumor infiltrating myeloid cells stimulated EGFR/MAPK signaling and increased the expression of PD-L1 on the epithelial cells to activate the PD-1/PD-L1 checkpoint (49). Depletion of myeloid cells by administration of Diphtheria Toxin (DT) to CD11b-diphtheria toxin receptor (DTR) mice reversed immune suppression and enabled CD8^+^ T cell activity, thus preventing PanIN formation in the iKras∗; CD11b-DTR GEMM and inhibiting tumor growth in CD11b-DTR mice transplanted with PDA cells (49). Therefore, controlled regulation of myeloid cells is an essential avenue for improvement of clinical efficacy against PDA.

### Myeloid-Derived Suppressor Cells in Cooperation With Tregs to Suppress T Cell Activation in PDA

MDSCs are Gr-1^+^CD11b^+^ myeloid cells that suppress T cell activation. Inducible nitric oxide synthase (64) enzyme in MDSCs regulates the release of nitric oxide from MDSCs, which subsequently causes DNA damage in CD8^+^ T cells (65). A recent study demonstrated that *in vivo* depletion of MDSCs led to a reduction in Tregs in pancreatic tumors (66). Through light sheet fluorescent microscopy and ex vivo functional assays, the authors showed that MDSCs induced Tregs by cell-cell direct interaction, which was lost in the Transwell system, and Tregs in turn affected the survival and/or proliferation of MDSCs (66). GM-CSF is necessary and sufficient to drive the development of Gr-1^+^CD11b^+^ cells (35, 36). GM-CSF blockade resulted in reduced MDSC infiltration and higher number of active CD8^+^ T cells in KPC tumors (36). Further, stroma-derived Dickkopf-1 (DKK1) activates β-catenin in MDSCs and regulates the recruitment and immunosuppressive effects of MDSCs (67). The two main categories of MDSCs are monocytic-MDSCs (Mo-MDSCs), characterized by the surface markers CD11b^+^Ly6G^-^Ly6C^Hi^, and granulocyte-derived MDSCs (Gr-MDSCs) by CD11b^+^Ly6G^+^Ly6C^Low^. Selective targeting of Gr-MDSCs was sufficient to induce the activation and proliferation of systemic and intratumoral CD8^+^ T cells (8).

CD11b^+^Ly6G^+^ cells are also identified as tumor associated neutrophils (TANs). In PDA, the abundance of TANs is strongly associated with poor prognosis (68). High level of CXCL5, a chemokine for the recruitment of neutrophils, as well as its receptor C-X-C chemokine receptor 2 (CXCR2), which is highly expressed on TANs, has been associated with poor outcome in PDA patients (68). CXCR2 inhibition prevented TAN accumulation in PDA TME, potently suppressed tumor growth and metastasis and sensitized PDA tumors to anti-PD-1 therapy (12, 13). The absence of TANs correlated with significant infiltration of activated T cells in the TME (13).Thus, inhibition of trafficking or depletion of MDSCs may offer a potential strategy to enhance the efficacy of immunotherapy for PDA. The role of CXCR2 ligands/CXCR2 biological axis in pancreatic cancer has been studied in a clinical trial (NCT00851955, results are pending). An ongoing clinical trial will evaluate the safety and tolerability of a CXCR1/CXCR2 inhibitor in combination with anti-PD1 as a maintenance therapy in patients with metastatic pancreatic ductal adenocarcinoma and will also assess the immunophenotypic and stromal changes to the tumor microenvironment after treatment (NCT04477343).

### Reprogramming of Tumor-Associated Macrophages in PDA as a Strategy to Restore Anti-Tumor Immune Responses

TAMs originate from inflammatory monocytes and tissue resident macrophages with different functions (69). Monocyte-derived TAMs function in antigen presentation whereas embryonically derived TAMs exhibit a pro-fibrotic transcriptional profile (9). TAMs can be recruited by cytokines and chemokines such as colony-stimulating factor-1 (CSF1), CCL2 and CCL5, and polarized into different states (70–76). Pro-inflammatory cytokines such as IFN-γ, TNF-α, and IL-12 secreted by classically activated M1-like macrophages recruit Th1 cells and stimulate anti-tumor activity (77), while alternatively activated M2-like TAMs produce anti-inflammatory cytokines such as IL-10 and TGF-β to promote the expansion of Th2/Th17 cells and Tregs (77–79). M2-like TAMs also produce Arg1 which in turn depletes L-arginine that is necessary for T cell function (80, 81). TAMs can exert opposite roles in cancer, either promoting or restraining tumorigenesis based on their polarization (82, 83). In PDA, TAMs have a highly dynamic and heterogeneous status, although as a whole they are inclined to be M2-like and have a profound influence on tumorigenesis and metastasis, as well as on immunosuppression and chemotherapeutic resistance. Currently, a growing interest in the field is to disrupt TAM recruitment or to reprogram TAMs to hinder tumor development, boost antitumor immunity and improve clinical therapy.

The CSF1 receptor (CSF1R) is expressed on F4/80^+^ macrophages and on monocytes in mice. Targeting TAMs through CSF1R inhibitor (CSF1Ri) or a CSF1 neutralizing antibody inhibited tumor growth, reduced metastatic burden and prolonged survival in KPC mice (84, 85). CSF1Ri treatment decreased the number of CD11b^+^Ly6G^-^Ly6C^Lo^F4/80^Hi^MHCII^+^ macrophages and CD11b^+^Ly6G^-^Ly6C^Hi^ Mo-MDSCs (86). The expression of immunosuppressive molecules, including *Pdcd1lg2*, *Il10*, *Arg1*, *Tgfb1*, and *Ccl22*, was reduced in macrophages while proinflammatory genes, such as *Il12a*, *Ifna*, *Ifnb1*, *Ifng*, *Cxcl10*, and *Nos2*, were upregulated, indicative of reprogramming of TAMs toward a M1-like phenotype (86). Consistently, CD3^+^CD8^+^ cytotoxic T cells and CD3^+^CD4^+^FOXP3^-^ effector T (Teff) cells were significantly up-regulated upon treatment with CSF1Ri, while CD4^+^FOXP3^+^ Tregs were down-regulated, ending up with a significantly improved Teff/Treg ratio (86). Ex vivo assays also revealed that CSF1 blockade alleviated immunosuppressive activities and enhanced antigen-presenting potential in both TAMs and DCs (86). Moreover, CSF1Ri upregulated PD-1 and CTLA-4 expression on T cells and sensitized PDA to immune checkpoint blockade. CSF1Ri-treated tumors also displayed less prominent αSMA^+^ stromal expansion, which was partly due to reduction of granulin, a secreted glycoprotein that stimulates fibroblast activation and migration. The expression of granulin is mediated by CSF1/CSF1R signaling in TAMs (87). CSF1R inhibitors Pexidartinib and Cabiralizumab have been tested in clinical trials with standard therapies or immune checkpoint blockade in advanced pancreatic cancer patients (NCT02777710, NCT03336216, NCT02526017). Although the toxicity of CSF1R inhibitor combined with immune checkpoint blockade was generally consistent with monotherapy, and the combination resulted in dose-related reduction of circulating monocytes^1^ (88), unfortunately, in phase II study (NCT03336216) the combination of Cabiralizumab and nivolumab (anti-PD1) with or without chemotherapy failed to improve progression-free survival of patients with advanced pancreatic cancer^2^. One possible reason of the unresponsiveness to these immunomodulatory approaches could still be the lack of active T cells in the ‘cold’ tumors. CSF1R inhibitor IMC-CS4 is currently being tested in combination with pancreatic cancer vaccine and immune checkpoint blockade in pancreatic cancer patients (NCT03153410).

Other approaches developed to reprogram TAMs include targeting Receptor-interacting serine/threonine protein kinase 1 (RIPK1), a critical receptor kinase on TAMs. Targeting RIPK1 with a small molecule GSK3145095 up-regulated STAT1 signaling in TAMs and reprogrammed intratumoral TAMs toward an MHCII^hi^TNFα^+^IFNγ^+^ immunogenic phenotype with a reduction in CD206, IL-10, TGF-β and Arg1 (89). RIPK1-inhibited TAMs induced cytotoxic T cell activation and the differentiation of T helper cells toward a mixed Th1/Th17 phenotype. RIPK1 inhibition thus led to active innate and adaptive immunity in both orthotopic KPC tumors and in organotypic models of human PDA. RIPK1 inhibition also synergized with anti-PD-1 treatment (89). However, the clinical trial of GSK3145095 was terminated because 50% of patients (4/8) involved in part 1 of this phase I/II study developed serious adverse events (NCT03681951).

While targeting TAMs emerges as a potential therapeutic strategy in pancreatic cancer, tumor-associated neutrophils might compensate for the loss of TAMs in PDA. Treatment with a CCR2 inhibitor to target CCR2^+^ TAMs resulted in a compensatory influx of CXCR2^+^ TANs in PDA patients (90). Accordingly, combination targeting of both CCR2^+^ TAMs and CXCR2^+^ TANs further augmented the anti-tumor immunity and enhanced the efficacy of chemotherapy in PDA. The CCR2/5 inhibitor BMS-813160 is under investigation in combination with chemotherapy or immune checkpoint blockade in advanced PDA patients (NCT03184870, NCT03767582).

### Dendritic Cell Scarcity and Insufficient T Cell Priming Contribute to the Cold Tumor of PDA

Increasing evidence points to the possibility that insufficient T cell priming due to lack of dendritic cells in PDA is a root cause of its nature as an immunologically cold tumor. Conventional dendritic cells (cDCs) have been recognized as one of the antigen-presenting cells that mediate T cell priming and cytotoxic T cell activity. A recent study showed that endogenous antigen-specific responses in PDA were aberrant due to a scarcity of DCs and an expansion of Th2/Th17 responses (91). Moreover, dysfunction of type 1 conventional dendritic cells (cDC1s) occurred in the earliest stages of tumorigenesis in KPC mice due to elevated apoptosis induced by IL-6 (92). Neutralization of IL-6 or combination treatment of CD40 agonist and Flt3 ligand rescued cDC1 abundance, leading to the control of tumor outgrowth (92). Restoring cDCs in KPC mice also blocked Th2 and Th17 cells and enhanced Th1 and CD8^+^ T cell activity, which ultimately resulted in reduced and lower-grade PanIN lesions accompanied by decreased collagen deposition and αSMA^+^ fibroblast density (91). Another study found that a distinct subset of DCs (CD11b^+^CD103^-^) predominated in PDA and induced tumor-promoting FOXP3^-^IL-10^+^IL-17^+^IFNγ^+^ regulatory CD4^+^ T cells through the secretion of IL-23 and TGF-β (93). This DC mediated-CD4^+^ T-cell differentiation was modulated by retinoic acid signaling (93).

Increasing attention has turned toward restoring T cell priming to overcome checkpoint unresponsiveness. CD40 is a cell surface molecule that regulates dendritic cells to promote T cell activation. CD40 on DCs binds to CD154 on CD4^+^ T helper cells and enables DCs to prime cytotoxic T cells (94). Activation of CD40 reprograms macrophages to destroy tumor stroma (95). Combination of CD40 activating antibody and anti-PD-1/CTLA-4 resulted in tumor regression and immunological memory in KPC mice (96). The T cell activating effect of this combination therapy was dependent on CD103^+^ DCs without the need for innate immune sensing pathways such as TLR, stimulator of interferon genes (STING) or interferon-α receptor (IFNAR) pathways, indicating that the CD40 pathway represents a distinct and alternative bridge between DCs and adaptive immunity in PDA (96). A previous clinical trial showed CD40 agonist monoclonal antibody (mAb) selicrelumab (formally named as CP-870,893 or RO7009789) with gemcitabine was well tolerated and therapeutic efficacy was observed in a cohort of patients with metastatic PDA (95) (NCT00711191). Another phase 1b study combining agonistic CD40 APX005M (sotigalimab) with gemcitabine plus nab-paclitaxel, with and without nivolumab, in 30 patients with metastatic PDA showed encouraging clinical activity and manageable toxicity (97). A recent phase 1b study used selicrelumab with or without gemcitabine and nab-paclitaxel in 16 resectable PDA patients prior to surgery followed by adjuvant chemotherapy and selicrelumab. The results showed CD40 agonist induced T cell immune response both at the tumor site and systemically in those early-stage PDA patients^3^. Currently, there’re more clinical trials exploring the combination of CD40 agonist with immune checkpoint blockade and/or chemotherapy in advanced pancreatic cancer (NCT03193190, NCT03329950).

In addition, there are encouraging results of dendritic cell-based immunotherapy to activate cytolytic T cell responses in pancreatic cancer from preclinical and clinical pilot studies (98, 99). In one study (NCT03114631), DCs generated from blood monocytes and pulsed with tumor lysates or tumor antigens MUC1 and WT1 were injected subcutaneously to 26 patients with stage II–IV pancreatic cancer. The preliminary results indicate DC-based immunotherapy is safe and provides immediate favorable outcome in pancreatic cancer patients (100). More clinical trials of DC vaccines in PDA patients are on-going (NCT04627246, NCT04157127, NCT03592888).

## B Cells Contribute to the Disfunction of T Cell-Dependent Antitumor Immune Responses in PDA

B cells are another immune cell population that plays a significant role in PDA progression, although some controversy regarding their precise function remains. Depletion of B cells using a CD20-specific mAb reduced PanIN formation in KC mice (101). An IL-35 expressing CD1d^hi^CD5^+^ B cell subset is required for the pro-tumorigenic effect of B cells in PDA (102). The growth of orthotopic KC cells in B cell-deficient (μMT) mice was significantly inhibited, a phenotype that was rescued by the reconstitution of CD1d^hi^CD5^+^ B cells through IL-35 mediated promotion of tumor cell proliferation (102). Bruton tyrosine kinase (BTK), a key B cell and macrophage kinase, contributes to the regulation of T cell-dependent anti-tumor immune responses in PDA (103). Phosphatidylinositol 3-kinase-gamma (PI3Kγ) activated BTK on B cells and Fc receptor γ-chain (FcRγ)^+^ TAMs, resulting in M2-type macrophage programming that suppressed CD8^+^ T cell cytotoxicity (103). BTK inhibitors Ibrutinib and ACP-196 were relatively well tolerated in metastatic PDA patients with the combination of chemotherapy or immune checkpoint blockade (NCT02403271, NCT02362048, NCT02562898). However, in the phase III trial patients with metastatic pancreatic cancer treated with Ibrutinib in combination with gemcitabine and nab-paclitaxel didn’t show improved progression free survival and overall survival (NCT02562898) (104). Besides, either monotherapy of ACP-196 or combined with pembrolizumab showed limited clinical activity in phase II study despite consistent reduction of MDSCs in peripheral blood (105). Only in two patients treated with combination therapy profound anti-tumor responses were observed, highlighting the necessity of targeting multiple TME components to improve efficacy as well as the need to better understand the complex human pancreatic tumor microenvironment, which may in part contributed to the failure of BTK inhibitors in this disease despite its success in hematologic malignancies (106).

## Extensive Network of Cancer-Associated Fibroblasts to Regulate Immune Suppression in PDA

CAFs are the major contributor to the desmoplastic stroma in PDA (107, 108). Extracellular matrix (ECM) and soluble factors secreted by CAFs are believed to activate key signaling pathways in cancer cells leading to cancer progression, cell survival, metastasis and drug resistance (107, 109, 110). ECM can also act as a physical barrier that prevents drug delivery (111). Sub-populations of CAFs have been noticed by several independent groups (112–115). A subpopulation of CAFs, myofibroblastic CAFs (myCAFs), are found adjacent to cancer cells. They have high expression of αSMA and have been hypothesized to restrict tumor progression. Inflammatory CAFs (iCAFs) are located in the desmoplastic stromal areas of the tumor. They express low level of αSMA but high levels of cytokines and chemokines such as IL-6, IL-11 and leukemia inhibitory factor (LIF) and promote tumor growth. A third sub-population of CAFs is antigen presenting CAFs (apCAFs), also described as mesothelial cells (116, 117). These CAFs express MHC class II related genes and can present antigens to CD4^+^ T cells. While these subpopulations of CAFs are spatially separated and phenotypically distinct, they still show some dynamic feature since myCAFs and iCAFs are interconvertible and apCAFs can also convert into myCAFs under certain conditions (113, 114).

**Figure 3:**
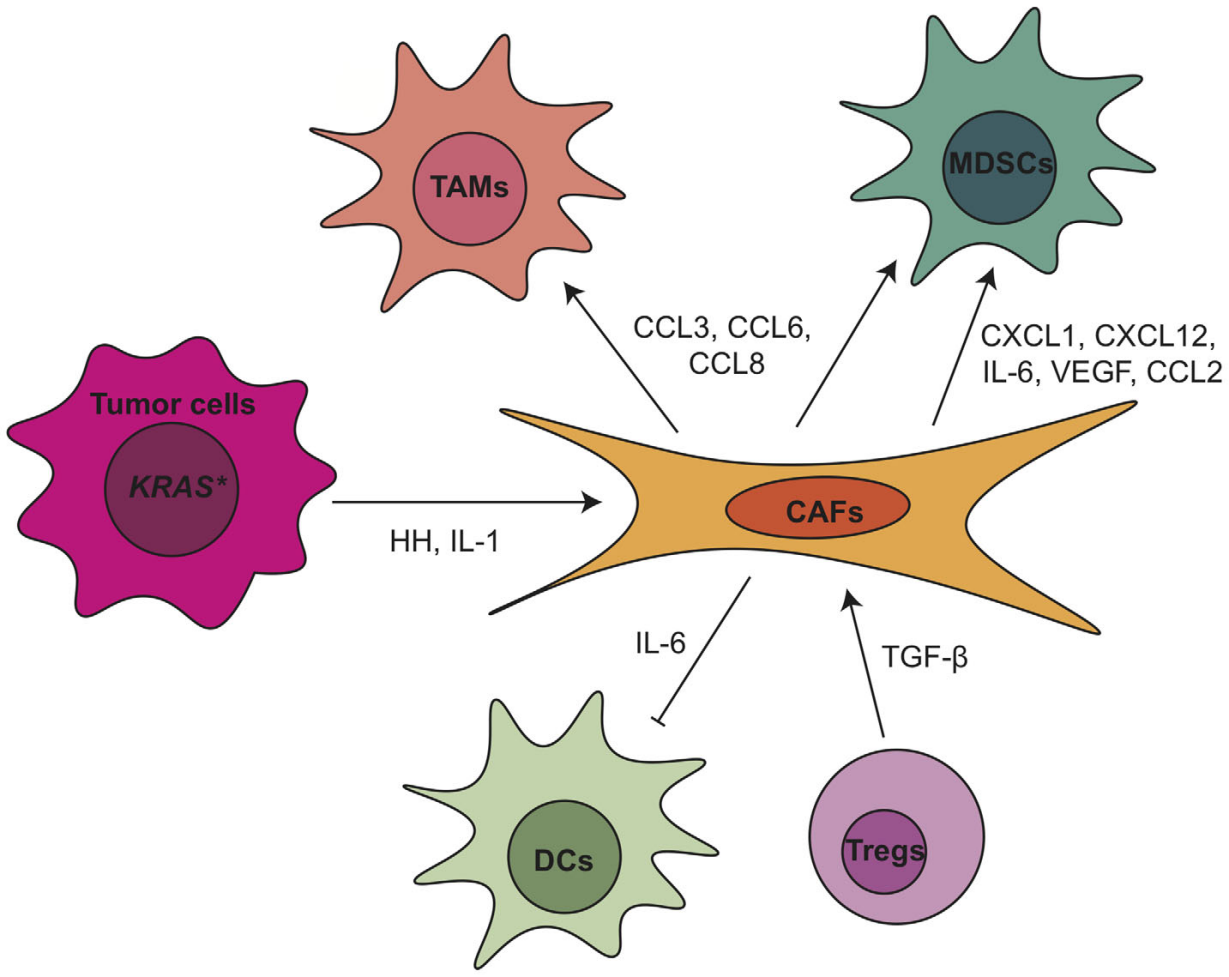
Crosstalk between tumor associated fibroblasts and other TME components within pancreatic cancer. CAF, cancer associated fibroblasts; CCL, C-C motif chemokine ligand; CXCL, C-X-C motif chemokine ligand; DC, dendritic cell; HH, hedgehog; IL, interleukin; MDSCs, myeloid-derived suppressor cells; TAMs, tumor associated macrophages; TGF-β, transforming growth factor β; Tregs, regulatory T cells; VEGF, vascular endothelial growth factor.

Due to the heterogeneity of CAFs, they play a complex role in the regulation of PDA progression and TME (**Figure 3**). Depletion of αSMA^+^ myofibroblasts starting at either the PanIN or the PDA stage led to invasive, undifferentiated, hypoxic tumors with diminished survival (118). Myofibroblast depletion also decreased overall immune infiltration in PDA but increased CD4^+^FOXP3^+^ Tregs, resulting in a reduction in both the Teff/Treg ratio and the cytotoxic CD8^+^/Treg ratio (118). A similar effect was observed when the Collagen 1 gene *Col1a1* was inactivated in a mouse model of pancreatic cancer (118). Due to the increased CTLA-4 expression following myofibroblast depletion, anti-CTLA4 immunotherapy reversed the disease acceleration caused by myofibroblast depletion and prolonged animal survival in *p48-Cre; LSL-Kras^G12D^; Tgfbr2^flox/flox^* (PKT) GEMM (118). On the other hand, the depletion of fibroblast activation protein (FAP)^+^ CAFs reduced the tumor growth and improved the efficacy of anti-CTLA-4 and anti-PD-L1 in KPC GEMM (6). FAP^+^ CAFs is the main source of CXCL12 in PDA, which coats and protects the cancer cells. Inhibiting CXCR4, a CXCL12 receptor, induced T cell accumulation among cancer cells and synergized with anti-PD-L1 to cause cancer regression (6). Pancreatic stellate cells (PSCs), characterized by lipid droplets in the cytoplasm, were found as a subset of pancreatic CAFs that correlates with increased suppressive immune cell populations and decreased T cells, natural killer (NK) cells, NK T cells and M1-type TAMs in the PDA tumor tissues (119, 120). The infiltration of CD8^+^ T cells was regulated through NFκB-mediated expression of CXCL12 in PSCs (121). PSCs also enhanced the differentiation and function of MDSCs through the production of MDSC-promoting cytokines IL-6, vascular endothelial growth factor (VEGF), CSF1 and chemokines CXCL12 and CCL2 (122). IL-6 secreted from PSCs led to the phosphorylation of STAT3 in peripheral blood mononuclear cells (PBMCs), which promoted the differentiation of PBMCs into MDSCs (122). Finally, when we inhibited Hedgehog (Hh) signaling, thus shifting the CAF population to a predominant iCAF phenotype, we also observed a decrease in cytotoxic T cells and an expansion of Tregs, indicating increased immunosuppression (123).

As mentioned above, CAFs secrete a variety of soluble factors that in turn shape the PDA TME, including IL-6, which plays multiple roles in the modulation of the immune response in PDA. Il6 not only is responsible for DC disfunction and MDSCs differentiation, as described earlier, but also regulates NK cell activity and Treg infiltration in PDA (124, 125). Combination blockade of IL-6 and PD-L1 led to increased Th1 T cell infiltration and decreased tumor growth (126). In iKras*; IL-6^-/-^ model, we observed deficiency of IL-6 resulted in reduction of tumor infiltrating macrophages and MDSCs (127). Clinical trials targeting IL-6 in PDA patients include IL-6 antibodies Tocilizumab and Siltuximab (NCT02767557, NCT04258150, NCT04191421). Other proteins secreted by CAFs as messenger to crosstalk with immune cells in PDA include but are not limited to CXCL1 (128), CXCL10 (129), IL-33 (130), ETS2 (131), galectin-1 (132), thymic stromal lymphopoietin (TSLP) (54) and βig-h3 (133).

The phenotype and function of CAFs in PDA TME is under control of epithelial cells as well as immune cells. For example, inactivation of oncogenic KRAS in the epithelial cells at the PanIN stage reduced α-SMA expression and inhibited CAF proliferation (14). Hedgehog ligands from tumor cells activated the Hh signaling in CAFs and promoted their expansion (134–136). Further study identified the Hh signaling effector glioma-associated oncogene homolog 1 (GLI1) as a critical transcriptional effector in this process (137). Deletion of a single allele of *Gli1* in iKras∗ GEMM was enough to disrupt the recruitment of immune cells by activated fibroblasts (137). Recently, our laboratory demonstrated that hedgehog signaling inhibition with smoothened antagonist LDE225 altered fibroblast composition with reduced myCAF and increased iCAF numbers in the KPC model (123). Immune cells, such as myeloid cells and Tregs, also participate in the regulation of CAFs. Stromal inactivation and remodeling of ECM were observed in both myeloid cell-depleted PanINs (49) and in CSF1Ri-treated PDA tumors (87). IL-1 and TGF-β have also been identified as ligands to promote CAF heterogeneity (125, 138). Recently, our lab showed that the loss of TGFβ1 upon Treg depletion reprogramed the fibroblast population with loss of αSMA^+^ myCAFs (62). Notably, TGFβ receptor inhibitor Galunisertib has been investigated in clinical trial in combination with durvalumab (anti-PD-L1) for metastatic PDA patients (NCT02734160). Newly published results of this trial showed phase II dose of galunisertib co-administered with durvalumab was tolerable and the disease control rate was 25% (8 patients had partial response or stable disease among 32 patients enrolled). The limited clinical benefit might be due in part to the aggressive nature of the advanced stage of disease (139). TGFβ receptor inhibitor PF-06952229 is currently under investigation in advanced solid tumors including pancreatic cancer patients (NCT03685591).

## Concluding Remarks

The stroma-rich, immunosuppressive microenvironment is a hallmark of pancreatic cancer. Tumor evasion of immune surveillance happens at the very early stages of tumorigenesis. Abundant immunosuppressive cells such as macrophages, Tregs and activated fibroblasts are evident even at the onset of acinar-ductal metaplasia (ADM), a key event for PDA initiation (140, 141). In contrast, antitumor effector cells such as CD8^+^ T cells are either scarce or excluded from the tumor nests. When intratumorally CD8^+^ T cells are present they are usually exhausted and express checkpoints such as TIGIT (11), lymphocyte-activation gene 3 (LAG-3) and PD-1 (32, 142). Recent research identified intratumoral exhausted T cells (PD-1^+^Lag3^+^Tox^+^) as induced by myeloid cell derived IL-27 in an orthotopic model of PDA (143). Those intratumoral T cells not only produced less IFNγ and Granzyme B but also expressed more IL-10, thus contributing to immune suppression in an autocrine manner. T cell exhaustion in cancer can be self-regulated through cell intrinsic mechanisms, however, the interaction between other cells or cytokines in the TME play an essential role in inducing T cell dysfunction. The TME in pancreatic cancer is composed of various types of cells that secrete abundant cytokines, including tumor cells, immunosuppressive cells, CAFs, inhibitory cytokines such as IL-6, IL-10 and TGF-β. The TME collectively form a complex and integrated immunosuppressive network to limit T cell differentiation, priming and drive T cell exhaustion. Therefore, when tumors have more CD8^+^ T cells they often also have increased granulocytes, immunosuppressive macrophages, and Tregs, and thus remain immune suppressive (32).

NK cells also play an important role in immune defense and immune regulation in cancer. In addition to their cytolytic activity, NK cells produce cytokines to modulate adaptive immune responses (144). In PDA, NK cells are reported being dysfunctional. NK cells from PDA patients exhibited a significant decrease in cytotoxic degranulation compared with those from healthy controls, a phenomenon that was associated with increased TGF-β1 expression in tumors (145). Future studies are needed to fully understand the mechanisms adopted by the TME to restrain NK cell activity in PDA, which might potentially provide new opportunities to devise new combination treatments for enhanced cancer immunotherapy response.

**Figure 4:**
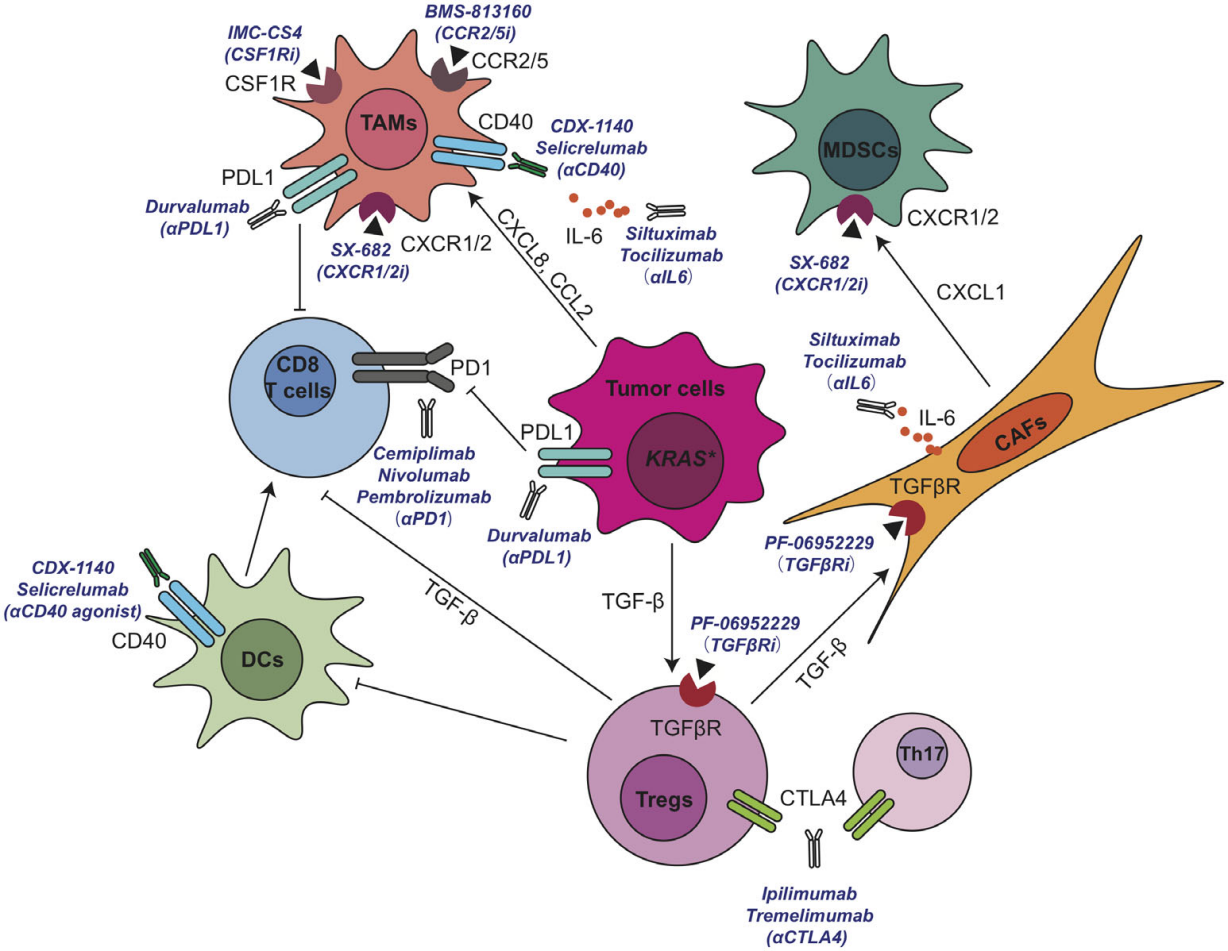
Current active clinical trials targeting immunosuppressive TME in pancreatic cancer. CAF, cancer associated fibroblasts; CCL, C-C motif chemokine ligand; CCR, C-C motif chemokine receptor; CSF1R, colony-stimulating factor 1 receptor; CXCL, C-X-C motif chemokine ligand; DC, dendritic cell; IL, interleukin; MDSCs, myeloid-derived suppressor cells; PD-1, anti-programmed cell death 1; PD-L1, programmed cell death ligand 1; TAMs, tumor associated macrophages; TGF-β, transforming growth factor β; Th, T helper; Tregs, regulatory T cells.

Significant progress has been made in the application of active immunotherapies including cytokines, immunomodulatory mAbs, and cancer vaccines or passive immunotherapies such as cell‐based therapies in cancer (146, 147). Mono-immunotherapies such as single immune checkpoint inhibitor anti-CTLA4 (ipilimumab), anti-PD1 (nivolumab, pembrolizumab) or anti-PD-L1 (duravalumab) have very limited benefits for PDA patients. It is now widely accepted that due to the complicated cellular crosstalk in PDA, targeting one immune-modulating pathway or a single population of stromal cells has very limited efficacy on reactivating immune system and restraining tumor progression. Therefore, simultaneously targeting multiple immunosuppressive components may acquire therapeutic benefits or improve the efficacy of immunomodulating anticancer therapeutics in PDA patients. In fact, a large number of clinical trials have explored the possibility of combination strategy such as the combination of multiple immunotherapy-based treatments, or combining immunotherapy with chemotherapy, radiation, and other cancer targeted therapies. So far, encouraging results from preclinical and clinical studies have demonstrated that combining an immunostimulatory approach, such as T cell priming *via* CD40 activation, with immune checkpoint blockade to prevent negative feedback signals on activated T cells represents the most promising treatment strategy to achieve clinical therapeutic benefit in this immunologically “cold” disease. We summarized a number of promising TME-targeting approaches for pancreatic cancer that are currently under clinical investigation in **Figure 4** and **Table 1**.

The advent of next-generation sequencing technology and large-scale tumor molecular profiling has shed light on the heterogeneous immune infiltration and tumor microenvironment in human PDA both across and within tumors and the heterogeneity in the expression levels of checkpoints on tumor infiltrating T cells (11, 148–150). Based on these considerations, it is important to understand the variety and individual differences in immune response for future translational studies and clinical trials, including personalized immunotherapy approaches.

With a better dissection of cell heterogeneity and their crosstalk involving cancer and stromal cells within the TME, strategies aimed at targeting multiple mechanisms with synergistic effects may sensitize PDA tumors to chemotherapy or immunotherapy through re-programing the tumor microenvironment of PDA.

## Author Contributions

WD wrote the manuscript. YZ and MP reviewed and edited the manuscript. All authors contributed to the article and approved the submitted version.

## Funding

This work was supported by NIH/NCI grants R50 CA232985 to YZ; R01CA151588, R01CA198074, and U01CA224145 to MP. This work was also supported by the University of Michigan Cancer Center support grant (P30CA046592), including an Administrative Supplement to MP.

## Conflict of Interest

The authors declare that the research was conducted in the absence of any commercial or financial relationships that could be construed as a potential conflict of interest.

## Footnotes

1
https://www.businesswire.com/news/home/20171109005417/en/Bristol-Myers-Squibb-and-Five-Prime-Present-Phase-1a1b-Data-Evaluating-Cabiralizumab-anti-CSF-1-receptor-antibody-with-Opdivo-nivolumab-in-Patients-with-Advanced-Solid-Tumors

2
https://www.businesswire.com/news/home/20200218005144/en/Five-Prime-Therapeutics-Provides-Update-on-Phase-2-Trial-of-Cabiralizumab-Combined-with-Opdivo®-in-Pancreatic-Cancer

3
https://www.abstractsonline.com/pp8/#!/9325/presentation/5136
